# Suicide prevention actions carried out by an academic psychiatry service in Rio de janeiro city

**DOI:** 10.1192/j.eurpsy.2022.2173

**Published:** 2022-09-01

**Authors:** J. Jaber

**Affiliations:** Clínica Jorge Jaber, Saúde Mental, Rio de Janeiro, Brazil

**Keywords:** suicidea ttemptin

## Abstract

**Introduction:**

Description of a structured work of primary prevention, based on a survey of the prevalence of suicidal behavior in the Brazilian population throughout life, performed by an academic service of psychiatry and chemical dependence. We describe a survey of the probability of suicide attempt in an academic internment service focused on psychiatry and drug addiction in the city of Rio de Janeiro.

**Objectives:**

Raise awareness of the need to call for help and 24-hour distress hotline phone outreach. Calculate a possible demand for mental health services to patients with severe suicidal behavior aiming at the necessary equipment to attend this population.

**Methods:**

Clarification actions through the press, development of a suicide prevention lecture program given in schools, surveillance cameras, military institutions, companies and laws, promotion of public events with music, activities, distribution of t-shirts, booklets and leaflets.Using the mental health care implementation system: identifying the patient, raising their needs and available resources, breaking resistance, advocating and treating, we raised in this institution that from January 01, 2019 to September 01, 2019, 137 patients were hospitalized with a serious suicide attempt.

**Results:**

According to a survey of the prevalence of suicidal behavior in the Brazilian population over the course of life, where out of 100 patients, 17 had suicidal thoughts, 5 planned, 3 attempted suicide and 1 was treated in the emergency room.

**Conclusions:**

The suicide prevention program has been very successful as the press promotes of the telephone number for immediate relief. His survey highlighted the need to create a specific suicide treatment and prevention program.

**Disclosure:**

No significant relationships.

